# Rationale, design, and methodology of a trial evaluating three models of care for HCV treatment among injection drug users on opioid agonist therapy

**DOI:** 10.1186/s12879-018-2964-5

**Published:** 2018-02-09

**Authors:** Matthew J. Akiyama, Linda Agyemang, Julia H. Arnsten, Moonseong Heo, Brianna L. Norton, Bruce R. Schackman, Benjamin P. Linas, Alain H. Litwin

**Affiliations:** 1 Department of Medicine, Montefiore Medical Center, Albert Einstein College of Medicine, Bronx, NY USA; 2 Department of Epidemiology and Population Health, Montefiore Medical Center, Albert Einstein College of Medicine, Bronx, NY USA; 3 Department of Healthcare Policy & Research, Weill Cornell Medical College, New York, NY USA; 4 Department of Epidemiology, Boston University School of Public Health, Boston, MA USA; 5 Department of Medicine, University of South Carolina School of Medicine–Greenville, Greenville, South Carolina USA; 6 Department of Medicine, Greenville Health System, Greenville, South Carolina USA; 7 Department of Medicine, Clemson University School of Health Research, Clemson, South Carolina USA

**Keywords:** Hepatitis C virus, Injection drug use, People who inject drugs, Directly observed therapy, Group treatment, Models of care

## Abstract

**Background:**

People who inject drugs (PWID) constitute 60% of the approximately 5 million people in the U.S. infected with hepatitis C virus (HCV). Treatment of PWID is complex due to addiction, mental illness, poverty, homelessness, lack of positive social support, poor adherence-related skills, low motivation and knowledge, and poor access to and trust in the health care system. New direct-acting antiviral medications are available for HCV with high cure rates and few side effects. The life expectancy and economic benefits of new HCV treatments will not be realized unless we determine optimal models of care for the majority of HCV-infected patients. The purpose of this study is to evaluate the effectiveness of directly observed therapy and group treatment compared with self-administered individual treatment in a large, urban opioid agonist therapy clinic setting in the Bronx, New York.

**Methods/design:**

In this randomized controlled trial 150 PWID with chronic HCV were recruited from opioid agonist treatment (OAT) clinics and randomized to one of three models of onsite HCV treatment in OAT: 1) modified directly observed therapy; 2) group treatment; or 3) control – self-administered individual treatment. Participants were age 18 or older, HCV genotype 1, English or Spanish speaking, treatment naïve (or treatment experienced after 12/3/14), willing to receive HCV treatment onsite, receiving methadone or buprenorphine at the medication window at least once per week, and able to provide informed consent. Outcomes of interest include adherence (as measured by self-report and electronic blister packs), HCV treatment completion, sustained virologic response, drug resistance, and cost-effectiveness.

**Discussion:**

This paper describes the design and rationale of a randomized controlled trial comparing three models of care for HCV therapy delivered in an opioid agonist treatment program. Our trial will be critical to rigorously identify models of care that result in high adherence and cure rates. Use of blister pack technology will help us determine the role of adherence in successful cure of HCV. Moreover, the trial methodology outlined here can serve as a template for the development of future programs and studies among HCV-infected drug users receiving opioid agonist therapy, as well as the cost-effectiveness of such programs.

**Trial registration:**

This trial was registered with ClinicalTrials.gov (NCT01857245). Trial registration was obtained prospectively on May 20th, 2013.

## Background

Hepatitis C virus (HCV) is the leading cause of cirrhosis, hepatocellular carcinoma, and liver transplantation in the United States. Successful HCV treatment leading to sustained virologic response (SVR) is associated with decreased progression of liver disease and increased survival [1]. Without active treatment, mortality and health care costs due to HCV are expected to increase over the next two decades [2].

People who inject drugs (PWID) constitute 60% of the approximately 5 million people in the U.S. infected with HCV [3]. Although a large proportion of PWID with HCV are willing to undergo treatment [4–6], few are offered HCV treatment due to concerns about suboptimal adherence rates [7, 8]. PWID face many challenges in adhering to therapy including addiction, mental illness, poverty, homelessness, lack of positive social support, poor adherence-related skills, low motivation and knowledge, and poor access to and trust in the health care system [9, 10]. New direct-acting antiviral (DAA) regimens are promising for treatment on this population. However, optimal cost-effective models of care that promote adherence and SVR have not been elucidated.

To maximize HCV treatment outcomes, we have developed a multidisciplinary model of HCV care that integrates substance abuse treatment, on-site primary care, and HCV-related care within opioid agonist treatment (OAT) clinics. Adherence support is particularly important for active drug users, because active drug use has been associated with non-adherence in the setting of HIV antiretroviral therapy (ART) [11–14]. Although treatment for addiction and access to multidisciplinary teams likely play important roles in successfully treating HCV-infected PWID, it is unknown which specific psychosocial and/or structural interventions are needed.

We have piloted two models of intensive care: modified directly observed therapy (mDOT), and group treatment (GT). The proposed mDOT and GT interventions are guided by Fisher’s Information-Motivation-Behavior Skills (IMB) model [15–17]. The IMB model asserts that information, motivation, and behavioral skills are fundamental determinants of adherence [15]. According to the IMB model, information and motivation work through behavioral skills to affect adherence. Behavioral skills are a critical prerequisite of adherence, and determine whether even well-informed and motivated individuals are able to adhere. The IMB model further specifies that personal and situational characteristics, such as poor psychologic health, substance abuse, unstable housing, or inadequate access to medical care, may moderate these relationships and impact adherence [15]. In extreme cases, strong negative effects on adherence are expected, and interventions aimed at improving information and motivation may not be effective without support.

In our mDOT model, adherence to HCV treatment is linked to an established behavioral skill, specifically clinic attendance addresses adherence skills: acquisition and administration of HCV medications; incorporation of HCV treatment into daily routines; coping with side effects (through side effect assessment and management); and acquisition of social support. While primarily operating on behavioral skills, the mDOT intervention also enhances both information and motivation (through support from nurses and other clinic staff).

In our GT model, patient groups initiate and complete HCV treatment in a weekly treatment group, which provides powerful social support to mitigate fears of side effects, promote efficient education, and deliver medications. GT addresses information (through education by providers), motivation (through peer and provider support), and behavioral skills (by dispensing medications during group). While mDOT and GT have been associated with good pilot outcomes [18], we do not know whether either model is more efficacious or cost-effective than the standard care.

The primary objective of this study is to determine the effectiveness of three models of care for HCV treatment in PWID. The aims of this study are 1) to determine whether mDOT or GT is more efficacious than a control group of self-administered individual treatment (SIT) for enhancing adherence and virological outcomes, and decreasing drug resistance; 2) to determine the incidence and factors associated with the development of drug resistance in PWID initiating HCV treatment; 3) to perform cost and cost-effectiveness analyses of each model of intensive on-site HCV care; 4) to determine whether adherence to HIV ART, HIV viral load (VL), and CD4 count are affected by initiating HCV therapy.

We hypothesize that 1) rates of adherence, treatment completion, and SVR will be significantly higher in the intensive intervention arms compared to the control arm, and that rates of resistance will be lower. We further hypothesize that the proportion of subjects in the intensive models of care arms who achieve an SVR will be equivalent to that observed in large registration trials. 2) We hypothesize that only a minority (~ 20%) of PWID will develop resistance, and that the relationship between adherence and resistance will be quadratic. 3) We hypothesize that the total cost of delivering the GT intervention will be lower than delivering the mDOT intervention, and that the incremental cost-effectiveness ratios for CGT compared with SIT and mDOT compared with SIT will each be less than $100,000/QALY. 4) We hypothesize that adherence to ART, HIV VLs, and CD4 counts will not be affected by initiating HCV therapy.

The trial entitled Intensive Models of HCV Care for Injection Drug Users (ClinicalTrials.gov NCT01857245) has completed enrollment as of 5/9/16, treatment as of 9/5/16, and follow up is ongoing. In this manuscript, we provide a detailed description of the trial design and the methodological approach to analysis, which will complement the reporting of the study findings.

## Methods/design

### Study setting

This study is a randomized controlled trial in which 150 PWID with chronic HCV (genotype 1) were recruited from three Division of Substance Abuse (DoSA) clinics. DoSA is a network of nine methadone maintenance clinics administered by the Albert Einstein College of Medicine in the Bronx, New York. DoSA clinics provide comprehensive substance abuse treatment including pharmacotherapy and supportive services to approximately 4200 adults (≥ 18 years) with current substance use disorders (typically heroin). The primary focus is treatment of opioid dependence with methadone, and the average methadone pick-up schedule is 5 times per week. Three of DoSA’s clinics were the sites for study recruitment and delivery of the SIT, mDOT and group interventions.

Multidisciplinary staff comprised of substance abuse counselors, nurses, a part-time social worker, and a medical team consisting of a physician and at least one physician assistant delivers substance abuse treatment at each DoSA clinic. The medical team provides comprehensive primary medical care, including HCV and HIV care.

### On-site HCV treatment program

HCV-infected patients with appropriate insurance (including Medicaid) are offered on-site HCV evaluation and treatment by primary care providers, and are free to choose whether to receive HCV and other medical care at the methadone clinic or elsewhere. Primary care providers receive ongoing HCV-related training from an internist with expertise in providing HCV treatment to drug users and follow standardized HCV treatment protocols [19].

This trial began when the standard of care was pegylated interferon (IFN) alfa-2a injections (180 mcg weekly), self-administered twice-daily ribavirin, and telaprevir or boceprevir. The trial was adapted to reflect the standard of care with the release of DAAs. Trial participants were initiated on HCV regimens from 10/29/13 to 5/23/16 according to AASLD guidelines current at the time of treatment initiation: telaprevir/pegylated interferon/ribavirin (TVR/IFN/RBV) as of 10/29/13, sofosbuvir/pegylated interferon/ribavirin (SOF/IFN/RBV) and sofosbuvir/ribavirin (SOF/RBV) as of 12/10/13, sofosbuvir/simeprevir (SOF/SMV) as of 8/11/14, and sofosbuvir/ledipasvir (SOF/LDV) as of 11/11/14.

### Trial inclusion and exclusion criteria

Potential trial subjects were eligible for inclusion if infected with HCV genotype 1, > 18 years of age, English or Spanish speaking, treatment naïve (or treatment experienced after 12/3/14), willing to receive HCV treatment onsite, and receiving methadone or buprenorphine at the medication window at least three times per week (or at least once per week after 6/26/15). Participants were excluded if they had known hypersensitivity to any study medication or were decompensated cirrhotic, unable or unwilling to provide informed consent, psychiatrically unstable, or pregnant or breast-feeding.

### Approvals and data safety and monitoring

The trial was approved by the Committee on Clinical Investigations of the Albert Einstein College of Medicine and the Institutional Review Board of Montefiore Medical Center. All participants provided written informed consent. We were testing a low-risk behavioral intervention that is highly integrated with usual clinical care; for this reason, we did not create an independent data safety and monitoring board. Instead we established a data safety and monitoring plan, which required interim analyses after every 50 patients enrolled to determine whether there were sufficient risks or benefits, or whether significant differences in virologic outcomes between study arms developed to warrant trial cessation.

### Recruitment

Medical providers were asked to invite all patients initiating on-site HCV treatment to consider enrolling in the trial. Subjects also self-referred after seeing flyers posted in clinics, or were referred by other subjects or through HCV support groups. The initial steps in subject recruitment included a brief screening survey, informed consent, and in depth verification of eligibility using medical records and discussion with providers.

### Randomization

**Fig. 1:**
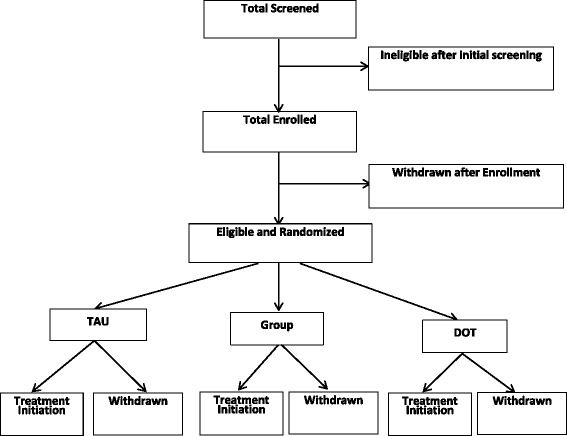
Flow chart of study recruitment and enrollment

We recruited 150 patients who were randomized to mDOT, GT, or in a 1:1:1 ratio in variable block sizes of 3–6 via central, computer-generated randomization (see Fig. 1). Two special randomization strategies (stratification and blocking) were used to avert imbalances in prognostic factors, and to ensure comparison groups of approximately equal size. We stratified randomization by IL28B genotype, HIV status, and stage of liver disease (cirrhosis vs. no cirrhosis) because of the importance of these factors in determining virologic outcomes [20–22]. To determine presence of cirrhosis, our algorithm included prioritizing a liver biopsy performed within the last 2 years (Ishak Stage 1–4 vs. 5/6) or any liver biopsy that demonstrated cirrhosis. As non-invasive testing became standard of care, we used fibrosure (< 0.75 vs ≥ 0.75), which was performed on all participants at baseline, and fibroscan (< 12.5 kPa vs ≥ 12.5 kPa) beginning 8/14/15 to determine the presence of cirrhosis [23, 24]. In cases where fibroscans and liver biopsies were not available and fibrosure testing was indeterminate, we used an AST to Platelet Ratio Index (APRI) score of ≥ 2.

### Models of care

All 3 models of care included co-located onsite care including HCV care, primary care, and OAT. Table 1 summarizes similarities and differences between the 3 models.

**Table 1:** Characteristics of three models of care

Element	SIT	mDOT	GT
Co-located care	X	X	X
Enhanced Social Support by providers		Nurse – individual	Medical provider – group
mDOT		1–6 doses/week	
Enhanced social support by patients			Peers – group
Enhanced education			Weekly educational session
Enhanced side effect management		Nurse – individual	Weekly group treatment

### Self-administered individual treatment (SIT)

Subjects randomized to SIT received all medications at the clinic from the clinic nurse, packaged in 7-day blister packs (oral medications). Medication were dispensed either weekly, biweekly, or monthly based on provider preference. In this arm, all medications are self-administered at home. For the subjects who received IFN early in the trial (10/29/13 – last date subject receiving IFN/SOF/RBV completed treatment), they were instructed on proper administration. The provider administered the first injection, and the subject administered the second injection under provider observation. The remainders of the doses were provided in a box containing a month’s supply of IFN.

### Modified DOT intervention arm (mDOT)

Subjects randomized to the mDOT arm received DOT during methadone visits. Since DOT of HCV medications was linked to methadone visits, the number of directly observed oral doses varied based on the number of days the patient attended the clinic to receive directly observed methadone (pick-up schedule). Depending on methadone pick-up schedule and dosing frequency, certain doses could not be observed (i.e., weekend doses, evening doses, and doses to be taken on a non-clinic day). In these instances, participants were given blister packs that include “take home doses,” for each unobserved dose, and were asked to return the blister pack at the next study visit, whether or not they took the pills. This intervention was considered modified DOT (mDOT) since initially only 3 to 6 out of 14 weekly TVR, 14 weekly RBV, and 7 weekly SOF, SMV, or LDV doses were observed. As subjects moved to once daily medications, subjects on more reduced pick-up schedules (once or twice weekly) were included within the trial as the frequency of reduced doses remained similar (14% - 29% observed doses for subjects on 1–2× weekly PUS for once daily regimens versus 21% observed doses for subjects on 3× PUS for twice daily regimens). Methadone clinic nurses notified clinicians when doses were declined, assessed for side effects, and referred subjects to onsite clinicians as necessary.

### Group treatment (GT)

We developed this model by adapting models linking HCV support with treatment [25–27] and models of group medical visits across many chronic conditions [28–30]. Subjects had orientation meetings, which were the first opportunity for subjects to meet as a group and interact with each other and the treatment team (physician and physician assistant). Subjects introduced themselves and shared concerns about HCV. The treatment team presented an overview of the HCV epidemic and its impact on drug users, natural history of HCV, and risks, benefits, and efficacy of HCV treatment. The group treatment protocol and schedule was discussed, and time was allowed for questions and discussion. Weekly CGT meetings had 6 components: 1) brief physical exams; 2) psychosocial support from peers and providers; 3) education; 4) side effect management; and 5) closing meditation on positive health. Six to 12 patients attended the groups, and entry in the groups occurred in a rolling fashion.

### Adherence measures

We used self-report and Med-ic® blister pack technology for RBV and DAA adherence in all three arms. Self-reported adherence was measured using a modified AIDS Clinical Trials questionnaire adapted for IFN, RBV and DAAs, as well as a single-item visual analogue scale (VAS) which has been shown to correlate well with both pill counts and virological outcomes in HIV-infected patients taking ART [31, 32]. Med-ic® is an innovative stick-on paper label for medication blisters that provides a disposable method to measure adherence [33]. Adherence is calculated by dose openings divided by total number of prescribed doses over thr different time intervals: 1) weekly adherence: patient receive credit if dose/s taken during the specified week; 2) daily: patient receives credit if dose/s taken during the specified day; 3) daily window adherence: patient receives credit if dose/s taken within the window period based on 25% of the dosing interval. For example a patient who is prescribed twice daily ribavirin 10 AM and 10 PM would get credit if morning dose were taken between 7 AM and 3 PM and evening dose was taken betweem 7 PM and 1 AM. A patient who is scheduled to take once daily medication at 10 AM would receive credit if dose taken between 4 AM and 4 PM. Treatment completion was defined as those who completed at least 80% of the duration of treatment.

### Virologic assessments

We obtained data on HCV VL tests performed at 4 weeks, 12 or 24 weeks (end of treatment response) through medical chart review. SVR was determined with a VL test 12 weeks after HCV treatment completion or discontinuation [19]. A VL test was also conducted at 24 weeks following treatment completion, which was the standard for assessing SVR prior to the introduction of DAAs [34]. Quantitative HCV VLs were performed using the Roche COBAS Ampliprep/Taqman assay, which quantifies HCV VL 43 IU/mL to 100 million IU/mL. After 10/9/14, we used COBAS Ampliprep/Taqman assay v2.0, which quantifies HCV VL 15 IU/mL to 100 million IU/mL. DoSA conducts annual HIV testing for all patients (unless they opt out) who are not already known to be HIV-positive. In addition, patients initiating on-site HCV treatment undergo HIV tests as part of our standard treatment protocol. For subjects without HCV VL tests in the chart, study sample was sent to the same laboratory for HCV VL testing.

### Psychosocial measures

Psychosocial domains and instruments are listed in Table 2. The full Audio Computer-Assisted Self-Interview (ACASI) takes between two and four hours to complete, and is administered at baseline. An abbreviated ACASI is administered at all other research visits.

**Table 2:** Schedule of research visits

Data Sources	Pre-Baseline	Baseline	Wk0	Wk4	Wk8	Wk12	Wk24	Final Tx Wk	FU Wk4	FU Wk12	FU Wk24
Blood Tests											
HCV Viral Load	X			X	X	X	X	X	X	X	X
Resistance Tests (NS3/4A, NS5A, NS5B Assays)		X		X				X	X	X	X
Other Clinical Measures											
Urine Toxicology (amphetamine, benzos, cocaine, methadone, opiates, oxycodone)		X		X	X	X	X	X	X	X	X
Methadone dose		X		X	X	X	X	X			
Chart Abstraction	X									X	
Blister Pack			X	X	X	X	X				
Questionnaires (*ACASI)*											
Demographics		X									
Adherence [32, 66, 67]				X	X	X	X	X			
ACTG for Interferon if used [31]				X	X	X	X	X			
Awareness of Viral Load questions				X	X	X	X	X		X	X
Quality of Life: EQ-5D-3 L [38, 68], HCV QoL [69, 70]		X		X	X	X	X	X		X	X
Depression: BDI-II [71, 72]		X		X	X	X	X	X		X	X
Healthcare Service Utilization (NMOS) [73]		X		X	X	X	X	X		X	X
Alcohol: AUDIT [74]		X		X	X	X	X	X		X	X
Drug and Alochol Use: ASI-Lite [75, 76]		X		X	X	X	X	X		X	X
Tobacco Questionnaire [77]		X									X
Social Support: Norbeck Social Support Questionnaire [78], MOS [79]		X				X	X	X		X	X
IMB Questions [80, 81]		X			X						
HCV Beliefs		X									X
Common Sense Model Questions [82]		X			X						
Psychiatric comorbidities: MINI [83]		X									
Trust in physician [84]		X									
Distrust in Health Care System [85]		X									
Trust in Health Care Provider [86]		X									
Subjective Opiate Withdrawal Scale [87]		X		X	X	X	X	X		X	X
Side Effects Survey		X		X	X	X	X	X			

### Cost

Costs of HCV treatment were collected by conducting chart reviews and assigning unit costs to all provider visits, lab tests, and medications based on Medicare fee schedules. The cost of the mDOT intervention was determined by conducting brief interviews with medical providers to estimate additional provider and patient time required for mDOT, using prevailing national wage rates to determine the cost of the time, and applying this cost to each DOT interaction. Patient time costs were valued based on the minimum wage, because most patients are likely to be unemployed. The cost of the GT intervention was similarly determined through activity logs that document the number of patients attending each session and the provider time per patient, as well as expense logs that were maintained for transportation and other incidental costs. Provider time spent on research activities, such as conducting research visit assessments, was excluded. We also examined the cost of health care services received outside of the study (emergency department visits, hospitalizations, substance abuse treatment) based on participant self-reports in the ACASI.

### Planned statistical analyses

The success of randomization will be checked by comparing key baseline covariates among the mDOT, GT, and SIT groups. If unbalanced covariates are identified, they will be adjusted for in the statistical analysis. We will apply Chi-square or Fisher’s exact tests to compare the proportion of subjects who achieve high overall adherence rate (≥ 80%), complete treatment, achieve SVR, and develop resistance in the 3 arms. If the omnibus equality test of adherence across the 3 groups is rejected, we will conduct the following 2 specific post-hoc pairwise tests with a Bonferroni adjusted 2-sided alpha = 0.025: mDOT vs. control, and GT vs. control. This strategy will be applied to multivariable logistic regressions to adjusted for the potential confounding variables not equally distributed between the study arms at baseline.

For the analysis of repeatedly measured adherence (using 6 post-baseline time points and adherence as a continuous measure), we will apply mixed effects linear model to test if the 3 groups are significantly different. The model will also include potential confounding variables. Again, if the omnibus equality test is rejected, we will conduct the 2 post-hoc pairwise tests described above. Likewise, we will apply mixed effects logistic regression to test the significance of mDOT and GT on repeatedly-measured undetectable HCV VL throughout the intervention period, adjusting for substance use. Changes in illicit drug use will be analyzed using urine toxicology data from each visit, counting the “person-month” as a unit of analysis, and analyzing the proportion of person-months that are positive for use of illicit drugs using a chi-square analysis.

As secondary analyses, we will examine if mDOT and GT effects, vs. SIT, will be mediated by IMB components. We will assess IMB model mediators of adherence at baseline, and during the intervention, and will analyze changes in those variables from the baseline to the intervention period. The indirect effects of IMB mediators will be tested by assessing changes in coefficients of the intervention effect (mDOT or GT) with and without IMB component(s) in mixed effects models. Confidence intervals of the mediated effects will be calculated by bootstrapping, and mediation assumed if confidence intervals do not include zero.

The proportion of participants who develop resistance will be determined with 95% confidence interval (CIs) calculated using exact binomial methods. The proportion of participants with resistance will then be determined within each of 4 groups determined by quartiles of adherence measured at the final time point in the study. We will apply multivariable exact logistic regression models to estimate odds ratios for resistance with the first quartile group as the referent.. To examine whether the relationship between adherence and resistance is linear, we will test a linearity of trends in odds ratios. If this linear trend is not significant, we will identify a quartile group that is associated with the highest odds of resistance.

#### Cost and cost-effectiveness analyses

To project future HCV costs and life expectancy, we will adapt the Hepatitis C Cost-Effectiveness model (HEP-CE), a Monte Carlo simulation model of HCV disease progression and treatment [35, 36]. The model simulates chronic HCV with fibrosis progression through each of the Metavir stages of liver fibrosis and decompensated cirrhosis. At all stages, HCV infection is associated with increased resource utilization and decreased quality of life (QOL) [37–39]. When HCV infection reaches the stage of cirrhosis (metavir F4), individuals begin to experience increased mortality attributable to liver disease [40]. With successful HCV therapy, disease progression halts, and mortality, resource utilization, and quality of life returns to that of HCV un-infected individuals [41]. HCV-related QOL is stratified by fibrosis stage, and costs of chronic HCV care (such as hospitalizations, ED visits, and clinic visits) are identified separately for patients with and without cirrhosis and stratified by age and sex.

We will populate the model with data collected by PREVAIL including cohort characteristics (demographics and fibrosis stage at baseline), treatment outcomes (rates of treatment toxicity and default, and proportion achieving SVR) and costs. We will explore the potential impact of resistance mutations by varying the efficacy of subsequent HCV retreatment for patients with resistance who choose to be retreated.

We will estimate the cost-effectiveness. of the 3 interventions: SIT, mDOT, and GT, assuming a lifetime horizon, health sector budgetary perspective, and 3% annual discounting to both costs and benefits [42]. We will interpret incremental cost-effectiveness ratios (ICER) assuming a $100,000/QALY willingness to pay threshold [43, 44]. To explore uncertainties in our results, we will perform one and 2-way sensitivity analyses on key input variables and probabilistic sensitivity analyses (PSAs) using second order Monte Carlo simulation on multiple variables [45].

## Discussion

This is the first RCT comparing two innovative HCV treatment strategies with self-administered individual treatment in an OAT program. This study will provide valuable information on optimal models of care to promote adherence and reduce viral resistance among PWID from effectiveness and cost perspectives. Understanding these factors will be crucial to addressing the HCV epidemic in this high-risk population.

There have been several operational challenges over the course of the trial due to rapid advances in HCV therapies from IFN-containing to IFN-free DAA regimens, as well as diagnostic modalities. These advances had several important implications for our study design and analysis. Since a heterogeneous study population would decrease our power to detect differences in SVR between treatment arms, we incorporated HIV positivity as a criterion for stratification in our randomization process. As efficacy became comparable with SOF-based regimens among HIV-infected and uninfected patients [46], HIV-positivity remained a criterion for stratification, which we will control for in our analysis.

Similarly, the IL28B genotype affected responses to HCV therapy in the IFN-era. Therefore, we also used IL28B (C/C vs. C/T or T/T) as a criterion for stratification. Since IL28B explained much of the variability in response between African Americans and non-African Americans [20, 47], and rates of SVR with triple therapy were shown to be equivalent in Latinos and non-Latino Whites [21], we did not stratify by race or ethnicity. We continue to stratify by IL28B; however, since the emergence of SOF-based regimens it is no longer a significant predictors of SVR. Thus, we do not anticipate this will be a factor influencing response to therapy within or between treatment arms.

Fibroscan technology became more widely available while the trial was being conducted. Fibroscan yields greater sensitivity and specificity to assess for severe fibrosis. We began using fibroscan technology to stratify our study participants on 8/14/15. We do not anticipate this will result in difference in stratification since fibroscans are widely comparable to liver biopsy and other non-invasive testing [48, 49].

Another advance that was introduced in this trial was electronic blister pack technology to monitor DAA adherence. In the IFN-era, strict adherence to IFN and RBV therapy was necessary to optimize SVR rates. Studies demonstrated that patients who adhere to at least 80% of the intended treatment schedule (taking at least 80% of the total dose of both IFN and ribavirin for at least 80% of the intended duration) were more likely to achieve an SVR [50–52]. Some studies suggested that ribavirin exposure was particularly important in determining response to HCV treatment; supporting the concept that adherence to daily oral medications required unique interventions [53, 54].

The study was originally powered (> 80%) to detect differences using SVR rates in the IFN era (with TVR or BOC). At that time, we anticipated that SVR rates in the mDOT and GT arms would be ≥70%, and ≤40% in the SIT arm [25, 55, 56]. With SOF-based regimens, which are taken orally daily, the effect-sizes are smaller; however, the latter are not currently known.

In the DAA era, due to the risk of viral resistance, adherence remains a critical issue [57]. Yet, there are currently no data describing associations between DAA adherence and virologic outcomes. Therefore, optimal adherence to DAA regimens is currently not known. Accurate measurement of adherence will be a critical issue to understand the direct effect of adherence on treatment failure. Previous RCTs have focused on DOT pegylated-IFN injections and RBV, but these trials have utilized diaries and pill counts for all medications [55, 58, 59]. We utilized electronic blister packs in our trial. This innovative technology will build on this literature by investigating adherence as measured by electronic monitors that have been shown to better predict virologic outcomes in HIV-infected population [60–62]. These results will be of particular relevance since two recent trials examined adherence to DAAs (as measured by diaries) among individuals who were on vs. not on OAT without active substance use [59], as well as active drug users on OAT [63]. While these studies demonstrated adherence rates were similar in these groups, the direct effect of adherence on virologic failure was not addressed.

This trial will also provide valuable information on the cost and cost-effectiveness of treating HCV in PWID. HCV treatment in PWID has been demonstrated to be cost-effective in various geographic settings [64, 65]. However, there is a paucity of data about the cost-effectiveness of various intensive models of care among PWID that could be used to inform implementation of these models. Given the high cost of DAAs and the importance of PWID as a driver of the HCV epidemic, the impact of findings about the cost-effectiveness of intensive models of care may yield information to guide policy recommendations for this critical population.

## Abbreviations

ACASI: Audio Computer-Assisted Self-Interview
APRI: AST to Platelet Ratio Index
BOC: Boceprevir
DAA: Direct Acting Antiviral
GT: Group treatment
HCV: Hepatitis C
IFN: Interferon
IMB: Information-Motivation-Behavior
MDOT: Modified Directly Observed Therapy
OAT: Opiate Agonist Therapy
PWID: People who inject drugs
RBV: Ribavirin
SIT: Self-administered Individual Treatment
SOF: Sofosbuvir
SVR: Sustained virologic response
TVR: Telaprevir
